# Reduced nitrogen availability in hydroponically grown Chinese broccoli does not affect photosynthetic performance and yield while enhancing nitrogen use efficiency and nutritional quality

**DOI:** 10.3389/fpls.2025.1745794

**Published:** 2026-01-21

**Authors:** Jie He, Wei Ling Tan, Lin Qin, Cheng-Hsiang Lai, Itamar Shenhar, Menachem Moshelion, Kee Woei Ng

**Affiliations:** 1 National Institute of Education, Nanyang Technological University, Singapore, Singapore; 2 Singapore-HUJ Alliance for Research and Enterprise (SHARE), Singapore, Singapore; 3 The Robert H. Smith Faculty of Agriculture, Rehovot, Israel; 4 School of Materials Science and Engineering, Nanyang Technological University, Singapore, Singapore; 5 Nanyang Environment and Water Research Institute (NEWRI), Singapore, Singapore

**Keywords:** Chinese broccoli, dietary minerals, nitrogen use efficiency, photosynthetic performance, phytonutrients, yield

## Abstract

Nitrogen (N) deficiency negatively affects the productivity and nutritional quality of leafy vegetables. N overfertilization leads to low nitrogen use efficiency (NUE), accelerates the eutrophication of water, and may reduce productivity and nutritional quality. In this study, hydroponically grown Chinese broccoli, Kai Lan, was supplied with a gradient of six N concentrations from deficient to surplus, ranging from 40 to 400 ppm. Compared to those grown with full N of 200 ppm, plants supplied with 40 and 400 ppm N had significantly lower yield and lower photosynthetic light use efficiency and CO_2_ assimilation rate. However, reduced N supply at 80, 120, and 160 ppm did not affect their photosynthetic performance and final yield. Nitrate (NO_3_^−^) and total reduced nitrogen (TRN) accumulation in plants increased linearly with increasing N supply from 40 to 200 ppm. NUE was the highest at 40 and the lowest at 400 ppm N. There was no difference in nitrogen harvest index among plants supplied with 80 to 400 ppm N, which was significantly higher than plants with 40 ppm N. Reduced or excessive N did not affect leaf total soluble protein and Rubisco proteins. Leaf total ascorbic acid (ASC) concentrations were significantly lower in plants supplied with 40 and 400 ppm N compared to the other plants. For leaf total phenolic compounds (TPCs), plants grown with 40 and 400 ppm N had the highest and lowest concentrations, respectively. N-deficient treatments with 80 to 160 ppm N resulted in increased accumulation of ASC and TPC as well as dietary mineral K compared to those grown with full 200 ppm N, while the opposite trend was observed for Fe. For Mg and Ca, plants grown with 40 to 160 ppm N had similar but significantly higher concentrations than those of plants grown with full N of 200 ppm and excessive N of 400 ppm. In conclusion, it is not necessary to supply Kai Lan with full N of 200 ppm, as even though they had lower NO_3_^−^ accumulation, higher nutritional quality is achieved without a yield penalty by reducing N to 120 ppm.

## Introduction

Brassicaceae vegetables are recognized for their rich source of dietary minerals, vitamins, and active bioactive compounds or phytonutrients, which have antioxidants and anticarcinogenic properties (Zhang et al., 2025). The consumption of Brassicaceae vegetables such as *Brassica oleracea* var. *alboglabra*, also known as Kai Lan, can reduce the risks of cancer and cardiovascular diseases (Jahangir et al., 2009). Moreover, Kai Lan, which has tender leaves and a thick, green succulent stem, is easy to grow with a short harvest lifespan. Although Kai Lai is a cool-season crop that grows well in temperate and subtropical regions, we have recently grown a Kai Lan hybrid (*B. alboglabra*, F1 hybrid—Lu Bao) healthily in a well-ventilated tropical greenhouse in Singapore (Harikumar et al., 2024), making it affordable worldwide. Along with its nutritional values, Kai Lan is a very popular leafy vegetable in Singapore and in many Asian cuisines.

Nitrogen (N) is essential for chlorophyll (Chl) production and synthesis of amino acids, proteins (Mu and Chen, 2021; Godoy et al., 2025), and many secondary metabolites (Amtmann and Armengaud, 2009). Photosynthetic performance, including light use efficiency (Godoy et al., 2025) and photosynthetic gas exchange (Evans and Clarke, 2019; Godoy et al., 2025), is highly correlated with leaf N concentrations. N deficiency causes low Rubisco concentration, resulting in decreased photosynthetic CO_2_ assimilation and reduced light energy usage. This can, ultimately, lead to excessive unutilized energy causing photoinhibition or photooxidation of photosynthesis (Godoy et al., 2025). NO_3_^−^ constitutes the most important form of N, which is required by the soilless culture of vegetable crops in large quantities. NO_3_^−^ availability can affect plant growth and development such as leaf area (Mu and Chen, 2021). In plants supplied with inadequate NO_3_^−^, the leaf area is significantly reduced with lower Chl content. Reduced Chl content decreases a plant’s photosynthetic capacity, resulting in lower shoot biomass (Mu and Chen, 2021). Moreover, photosynthetic light use efficiency and gas exchange are also affected by reduced NO_3_^−^ availability (He et al., 2011a, 2014; Mu and Chen, 2021). Limited NO_3_^−^ promotes the biosynthesis of abscisic acid (ABA), leading to stomata closure (Mu et al., 2018). In addition, K concentration is lowered in plants supplied with limited N. This decreases stomatal conductance, as K is required for regulating the opening and closing of the stomata (Tränkner et al., 2018). However, low N supply increases the accumulation of ASC (Zhang et al., 2016) and TPC (Kováčik et al., 2007). Plant root systems serve as the primary interface between plants and the growth medium. NO_3_^−^ availability influences the morphology and architecture of roots, including their total root length, number of root tips, surface area, and diameter, which in turn, determines the ability of plants to acquire N (Sun et al., 2017; Liu et al., 2020; Pélissier et al., 2021; Lopez et al., 2023). On the other hand, in plant nutrition, different mineral nutrients interact in a direct and/or indirect manner. The deficiency of one element can influence the proper uptake of others and their use efficiency (Romera et al., 2021).

Due to the relatively low cost of NO_3_^−^ fertilizer, overfertilization of NO_3_^−^ to ensure yield has been a common issue in crop production (Fageria and Baligar, 2005; Dong and Lin, 2020). A surplus of NO_3_^−^ supply to plants leads to lower crop yield, as excessive NO_3_^−^ supply can be detrimental to plant growth (Chen et al., 2004; Dong and Lin, 2020). For instance, a high concentration of NO_3_^−^ supply to plants suppressed lateral root formation, root hair development, and root elongation (Okushima et al., 2011). Some research has suggested that high NO_3_^−^ supply to leafy vegetables such as rape (*B. campestris* L.), Chinese cabbage (*B. chinensis* var. *oleifera* Makino et Nenoto), and spinach (*Spinacia oleracea* L.) could even be detrimental to whole plant development (Chen et al., 2004). Excessive NO_3_^−^ could result in the decline in photosynthetic efficiency due to photodamage of photosystem II (PSII) oxygen-evolving complex or inactivation of Rubisco (Cun et al., 2021). It was reported that with excessive NO_3_^−^ supply to vegetables, the synthesis of both ASC (Mozafar, 1993; Lee and Kader, 2000; Stefanelli et al., 2010) and TPC (Elhanafi et al., 2019) was negatively affected. Studies have shown that overfertilization of NO_3_^−^ not only leads to low nitrogen use efficiency (NUE) and accelerates the eutrophication of water but also results in excessive accumulation of NO_3_^−^ in vegetable crops (Congreves et al., 2021; Neocleous et al., 2021; Valenzuela, 2024). NO_3_^−^ is generally of low toxicity, but conversion to NO_2_^–^ results in interaction with hemoglobin. This affects oxygen transport, leading to methemoglobinemia in infants (Hegesh and Shiloah, 1982) and possibly gastric cancer as well as other diseases (Bryan et al., 2012). Moreover, excessive use of NO_3_^−^ increases NO_3_^−^ runoff in water bodies, resulting in eutrophication, depleting oxygen in water, harming aquatic life, and causing environmental pollution (Fageria and Baligar, 2005). Therefore, it is important to manage the amount of NO_3_^−^ supply to vegetable crops in order to maintain crop yield and nutritional quality while minimizing environmental pollution.

In our previous study with the same Kai Lan hybrid (*B. alboglabra*, F1 hybrid—Lu Bao) used in this study, which was supplied with nutrient solution containing 20, 40, 80, 120, 160, 200, 400, and 800 ppm N (Harikumar et al., 2024), it was found that plants supplied with severe N limitations of 20 and 40 ppm had lower yield compared those grown with higher N concentrations of 80, 120, 160, 200, 400, and 800 ppm. Furthermore, Kai Lan plants supplied with 400 and 800 ppm of N did not show further increase in final yield compared to those supplied with 120, 160, and 200 ppm N. Accumulation of NO_3_^−^ in the leaves increased linearly with increasing N supply from 20 to 160 ppm, with a maximum accumulation from 160 to 200 ppm, and then decreased from 400 to 800 ppm N (Harikumar et al., 2024). These results agree with the fact that hydroponic plants can withstand concentrations of N up to approximately 200 ppm, which is the full concentration. Most of the studies on N availability effects on growth and the accumulation of NO_3_^−^ were carried out with salad vegetables such as lettuce under different NO_3_^−^ levels. This study was conducted with the popular Chinese leafy vegetable Kai Lan, supplied with a gradient of six N concentrations of 40, 80, 120, 160, 200, and 400 ppm from deficiency to surplus. According to Hoagland’s solution recipe design, 200 ppm N is required for a full strength of hydroponic solution. This project aimed to investigate the impacts of N availability on NUE and NO_3_^−^ accumulation in roots, stems, and leaves in comparison to full N of 200 ppm. As the increase in photosynthetic activity can have an important role in reducing NO_3_^−^ accumulation, enhancing productivity, and improving nutritional quality (Godoy et al., 2025), this study also aimed to investigate the impacts of N availability on NUE; NO_3_^−^ accumulation in the roots, stem, and leaves; and photosynthetic activity and nutritional quality of Kai Lan plants. This study would provide farmers with information on the optimal N supply to Kai Lan plants to enhance their yield and nutritional quality. In addition, supplying Kai Lan plants with the optimal concentration of N increases NUE, prevents environmental pollution, and promotes sustainable practices.

## Materials and methods

### Plant materials and experimental design

Seeds of Kai Lan (*B. alboglabra*, F1 hybrid—Lu Bao) were germinated on moist filter paper in petri dishes. After germination, the seedlings were inserted into polyurethane cubes in trays filled with water. All seedlings were placed along the corridor under natural sunlight with an average maximal photosynthetic photon flux density (PPFD) of 200 μmol m^−2^ s^−1^. After 7 days, the seedlings were then transplanted into hydroponic systems in the greenhouse. They were supplied with nutrient solutions of six different N concentrations of 40, 80, 120, 160, 200, and 400 ppm. The recipe of the nutrient formulation has been provided in our recently published article on *Amaranthus dubius* (Chinese spinach) (Harikumar et al., 2025), and it is also found in the **Supplementary Materials** (**Supplementary Table S1**). All plants were grown under an average maximal PPFD of 600 to 800 μmol m^−2^ s^−1^ during midday on sunny days, with ambient temperature ranging from 23°C to 39°C. The minimal and maximal ambient relative humidity were approximately 33% (day) and 98% (night). The average pH values of the nutrient solution for plants grown between 40 and 400 ppm N were between 6.0 and 6.5. The electrical conductivities were monitored every other day and maintained at 1.2, 1.4, 1.7, 2.0, 2.2, and 3.0 mS cm^−1^ for the nutrient solutions 40, 80, 120, 160, 200, and 400 ppm N, respectively.

### Measurements of leaf growth and shoot and root productivity

Five plants from each treatment were harvested after 5 weeks of transplanting. Total leaf number was recorded. Leaves, stems (leaves and stems = shoots), and roots were separated for fresh weight (FW, g) measurements. Total leaf area (TLA, cm) was measured using a leaf area meter (WinDIAS3 Image Analysis system). Leaves, stems, and roots were then dried at 80 °C for 4 days, before reweighing them to obtain dry weight (DW, g). Specific leaf area (SLA) was determined as *L*_a_/*L*_DW_, where *L*_a_ = leaf area (cm^2^) and *L*_DW_ = leaf DW (g).

### Analysis of root morphology

Four plants from each treatment were harvested after 5 weeks of transplanting. The roots of a Kai Lan plant were spread out and placed in a tray of water and were scanned using the WIN MAC RHIZO scanner. Afterward, the WIN MAC RHIZO V 3.9 program was used to analyze the root morphological parameters including total root length (cm), total number of root tips, total root surface area (cm^2^), and average root diameter (mm).

### Determination of chlorophyll and carotenoids

Fresh leaf discs of 0.05 g were obtained from four different fully expanded youngest leaves of four different plants under each treatment. The leaf discs were soaked in 5 mL of N,N-dimethylformamide (N,N-DMF, Sigma chemical co., Singapore.) in darkness for 48 h at 4°C. A spectrophotometer (UV-2550 Shimadzu, Kyoto, Japan) was used to measure the absorption of Chl a, Chl b, and carotenoids (Car) at 647, 664, and 480 nm, respectively. Chl and Car concentrations (μg g^−1^ FW) were calculated according to Welburn (1994).

### Measurement of Chl fluorescence F_v_/F_m_ ratio

Maximum quantum yield of PSII was estimated in dark-adapted fully expanded eight youngest leaves inside the greenhouse by Chl fluorescence F_v_/F_m_ ratio between 09:00 and 10:00 a.m. (defined as morning F_v_/F_m_ ratio) and between 13:00 and 14:00 p.m. (defined as midday F_v_/F_m_ ratio) using the Plant Efficiency Analyzer (Hansatech Instruments, UK) according to He et al. (2011b).

### Measurements of electron transport rate, effective quantum yield of PSII, photochemical quenching, and non-photochemical quenching

The youngest fully expanded leaves were harvested between 09:00 and 09:30 a.m. Four-leaf discs were obtained from four different leaves of four different plants under each treatment. The leaf discs were placed on moist filter papers in petri dishes in the laboratory at 25°C. They were pre-darkened for 20 min prior to measurements. Via the IMAGING PAM MAXI (Walz, Effeltrich, Germany), images of fluorescence emission were digitized within the camera and transmitted via a Firewire interface (400 megabits/s) (Firewire-1394, Austin, TX, USA) to a personal computer for storage and analysis. Measurements and calculations of electron transport rate (ETR, µmol electron m^−2^ s^−1^), effective quantum yield of PSII (ΔF*/*F_m_′), photochemical quenching (qP), and non-photochemical quenching (NPQ) were determined according to He et al. (2011b).

### Measurements of light-saturated photosynthetic CO_2_ assimilation rate, stomatal conductance, internal CO_2_ concentration, and transpiration

Measurements of assimilation rate (*A*_sat_), stomatal conductance (*g*_s sat_), internal CO_2_ concentration (*C*_i_), and transpiration (*T*_r_) were carried out from the youngest fully expanded leaves between 09:00 and 11:00 a.m. in the greenhouse with an open infrared gas analysis system with a 6-cm^2^ chamber (LI-COR Portable Photosynthetic System, LI-6400, Biosciences, South San Francisco, CA, USA). Readings were taken with an LED light source, which supplied 1,000 μmol m^−2^ s^−1^ of PPFD with wavelengths ranging from 420 to 510 nm and 610 to 730 nm. The average CO_2_ concentration, temperature, and relative humidity in the leaf chamber were 433 ± 4 μmol·mol^−1^, 32 °C ± 1°C, and 62.4% ± 6%, respectively. For each treatment, four measurements were made from four different leaves of four different plants.

### Determination of NO_3_^−^

Dried plant tissues of 0.01 g from four different plants were ground with 10 mL of deionized water before incubating at 37°C for 2 h. Sample turbidity was then removed by filtering the mixture through a 0.45-μm pore diameter membrane via a vacuum filter. The final volume was made to 50 mL. NO_3_^−^ concentration of the plant tissue was determined using the Flow Injection Analyzer (Model Quikchem 8000, Lachat Instruments Inc., Milwaukee, USA) according to He et al. (2020).

### Determination of TRN

Dried plant tissues of 0.05 g from four different plants were digested using a Kjeldahl tablet and 5 mL of concentrated sulfuric acid for 60 min at 350°C following the method of Allen (1989). The mixture was allowed to cool before the TRN concentration was determined using a Kjeltec KT8400 analyzer (Foss Tecator AB, Höganäs, Sweden) through titration.

### Determination of leaf total soluble protein and Rubisco protein by SDS-PAGE

Fresh leaves of 1 g from four different plants were ground before adding to 6 mL of extraction buffer (He et al., 2017). The mixture was centrifuged at 35,000 rpm for 30 min at 4°C (Beckman ultracentrifuge Optima XL-100K). A total of 1 mL of the supernatant was added to 4 mL of 80% cold acetone and centrifuged for 10 min at 4,000 rpm, using a bench-top centrifuge. Total soluble protein (TSP) was determined according to He et al. (2017). Protein extract was diluted (1:1 ratio) with solubilizing solution before loading onto a pre-cast gradient gel (PROTEAN TGX precast gel, any KD, Bio-Rad, USA). Electrophoresis was performed under constant voltage. The gel was then stained in Coomassie brilliant blue and destained with 7% acetic acid and 25% ethanol. The FluorChem 8800 gel imaging system was used to analyze the resultant bands under visible light according to He et al. (2017).

### Nitrogen use efficiency and nitrogen harvest index

NUE was calculated by dividing the crop yield (Y) by the N inputs, NUE = Y ÷ N, yield per unit of fertilizer N applied (Congreves et al., 2021). Nitrogen harvest index (NHI) was defined as the ratio of N in the harvestable product (shoot) divided by the total crop N. It identifies the amount of N translocation (in percentage) to the economic component (yield) (Congreves et al., 2021).

### Determination of ASC

Fresh leaves of 1 g from four different plants were extracted in 5 mL of ice-cold 2% (w/v) metaphosphoric acid. The homogenate was centrifuged at 4°C and at 9,000 rpm for 40 min. A supernatant aliquot of 0.3 mL was added to 0.2 mL of 45% (w/v) K_2_HPO_4_ and 0.1 mL of 0.1% (w/v) homocysteine. The mixture was incubated for 15 min at 25°C before adding 1 mL of 2 M citrate–phosphate buffer (pH 2.3) and 1 mL of 0.003% (w/v) 2,6-dichlorophenolindophenol (DCPIP). A spectrophotometer (UV-2550 Shimadzu, Kyoto, Japan) was used to measure the absorbance at 524 nm (Leipner et al., 1997).

### Determination of TPC

Fresh leaves of 1 g from four different plants were added to 10 mL of 80% methanol. The extracts were shaken for 30 min at 200 rpm and centrifuged for 20 min at 3,500 rpm. A total of 0.5 mL of supernatant was diluted with 0.5 mL of diluted Folin–Ciocalteau reagent and 1 mL of 7.5% Na_2_CO_3_. After 20 min, a spectrophotometer (UV-2550 Shimadzu, Kyoto, Japan) was used to measure the absorbance at 765 nm, according to Ragaee et al. (2006).

### Measurements of dietary minerals

Dried tissues of 0.2 g from four different plants were microwave-digested in 4 mL of 65% nitric acid using the UltraWAVE single reaction chamber microwave digestion system (Milestone, Inc., Shelton, CT, USA) before diluting with Milli-Q water to a final volume of 25 mL. The Optima 8300 ICP-OES (inductively coupled plasma optical emission spectrophotometer) and WinLab 32 (Perkin Elmer, Waltham, MA, USA) were used to measure and calculate the dietary mineral concentrations of K, Ca, Mg, and Fe.

### Statistical analysis

Data analysis was performed using RStudio (RStudio Team, 2020) for the statistical analysis. In all multiple comparison tests, data were checked for normal distribution using the Shapiro–Wilk test and homogeneity of variance using Levene’s test. One-way analysis of variance (ANOVA) and Tukey’s multiple comparison tests were then carried out to discriminate among the means of the different groups, where *p* < 0.05 indicated that the means were significantly different.

## Results

### Leaf growth and root and shoot productivity

**Figure 1:**
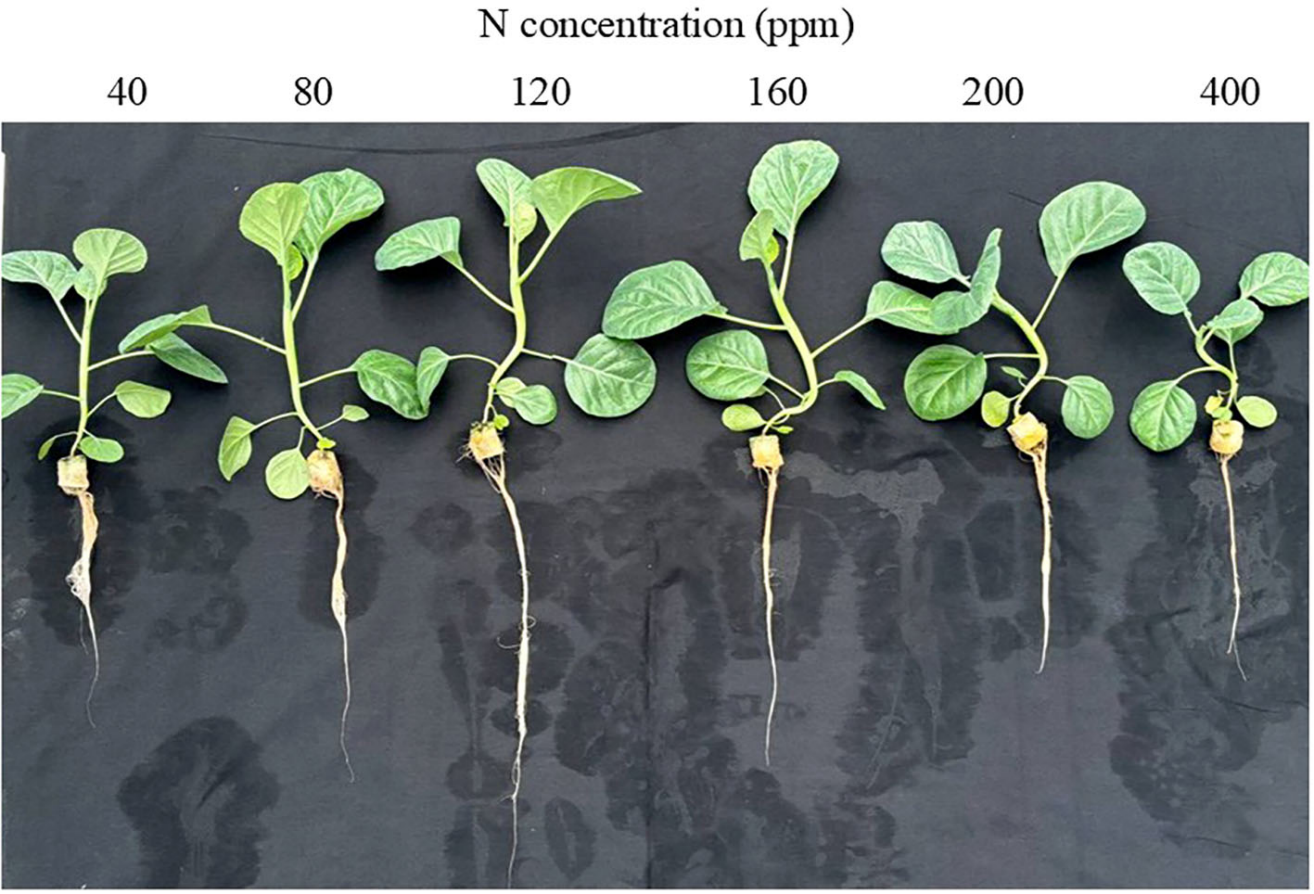
Kai Lan plants grown with different N concentrations at harvest (5 weeks after transplanting).

**Figure 2:**
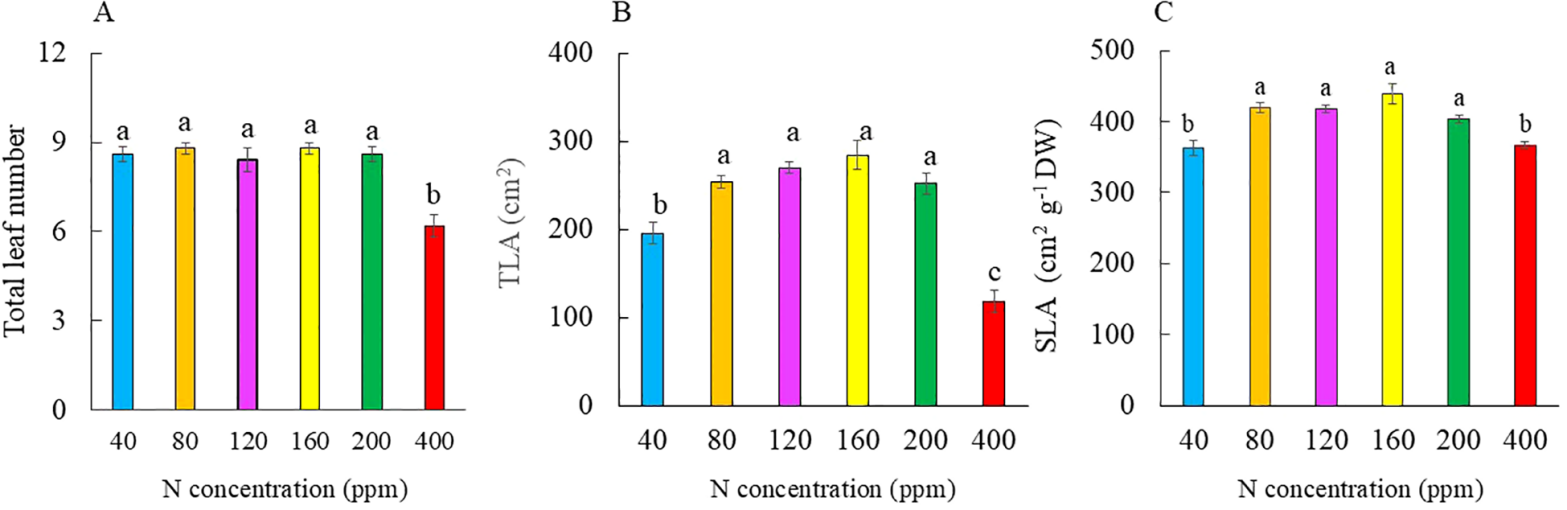
Total leaf number **(A)**, total leaf area (TLA) **(B)**, and specific leaf area (SLA) **(C)** of Kai Lan plants grown with different N concentrations at harvest (5 weeks after transplanting). Values are means (± S.E.) where different letters indicate significant differences (*p* < 0.05) of five replicates as determined by Tukey’s multiple comparison tests.

**Figure 3:**
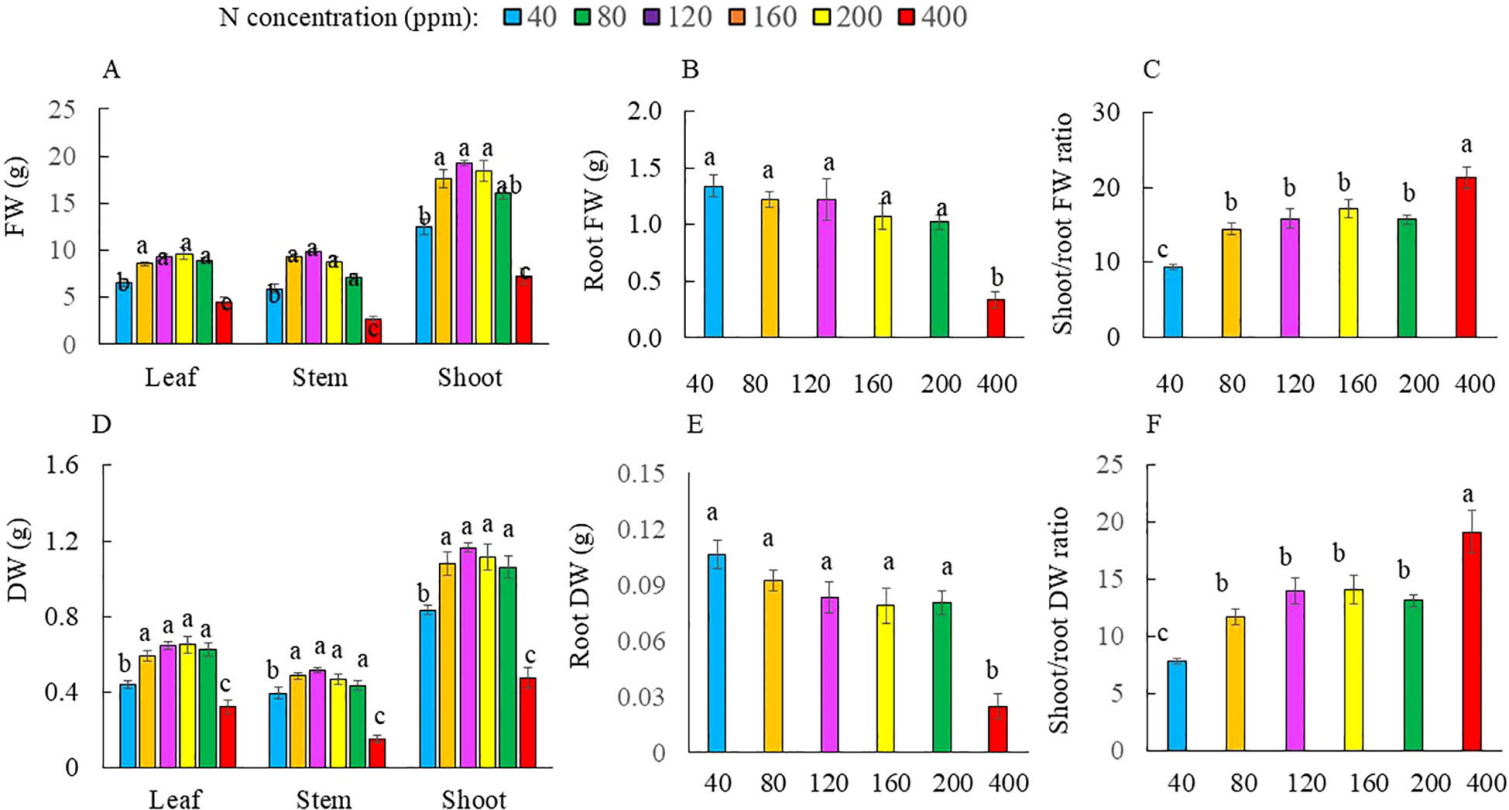
Leaf FW, stem FW, shoot FW **(A)**, root FW **(B)**, shoot/root FW ratio **(C)**, leaf DW, stem DW and shoot DW **(D)**, root DW **(E)**, shoot/root DW ratio **(F)** of Kai Lan plants grown with different NO_3_^−^ concentrations at harvest (5 weeks after transplanting). Values are means (±S.E.)

**Figure 1** shows the Kai Lan plants grown with different N concentrations at harvest (5 weeks after transplanting). Grown with 40 and 400 ppm N, Kai Lan plants were much smaller compared to the other N treatments. At harvest, there was no significant difference in the total leaf number of plants supplied with 40 to 200 ppm N. Plants grown with 400 ppm N, however, had significantly lower total leaf number compared to all the other plants (**Figure 2A**). While there was no significant difference in TLA among Kai Lan plants supplied with 80 to 200 ppm N, the TLA of plants grown with 40 ppm N was smaller than plants with 80, 120, and 160 ppm N, but greater than plants with 400 ppm N (**Figure 2B**). For Kai Lan plants grown with 80 to 200 ppm N, there was no significant difference in SLA. The SLA of plants grown with 40 and 400 ppm N was significantly smaller than plants grown with 80 to 200 ppm N (**Figure 2C**). Kai Lan plants grown with 400 ppm N had the lowest yield, with the lowest FW and DW of the leaves, stems, and shoots. There were no significant differences in these parameters among plants grown with 80 to 200 ppm N, which were significantly higher than those grown with 40 ppm N (**Figures 3A, D**). However, the FW and DW of the roots were similar in plants grown with 40 to 200 ppm N, which was significantly higher than that of plants grown with 400 ppm N (**Figures 3B, E**). Both shoot/root FW and DW ratios were significantly higher in plants supplied with 400 ppm N, while they were significantly lower in plants grown with 40 ppm N. There were no significant differences in shoot/root FW and DW ratios among the other treatments (**Figures 3C, F**).

### Root morphology

**Figure 4:**
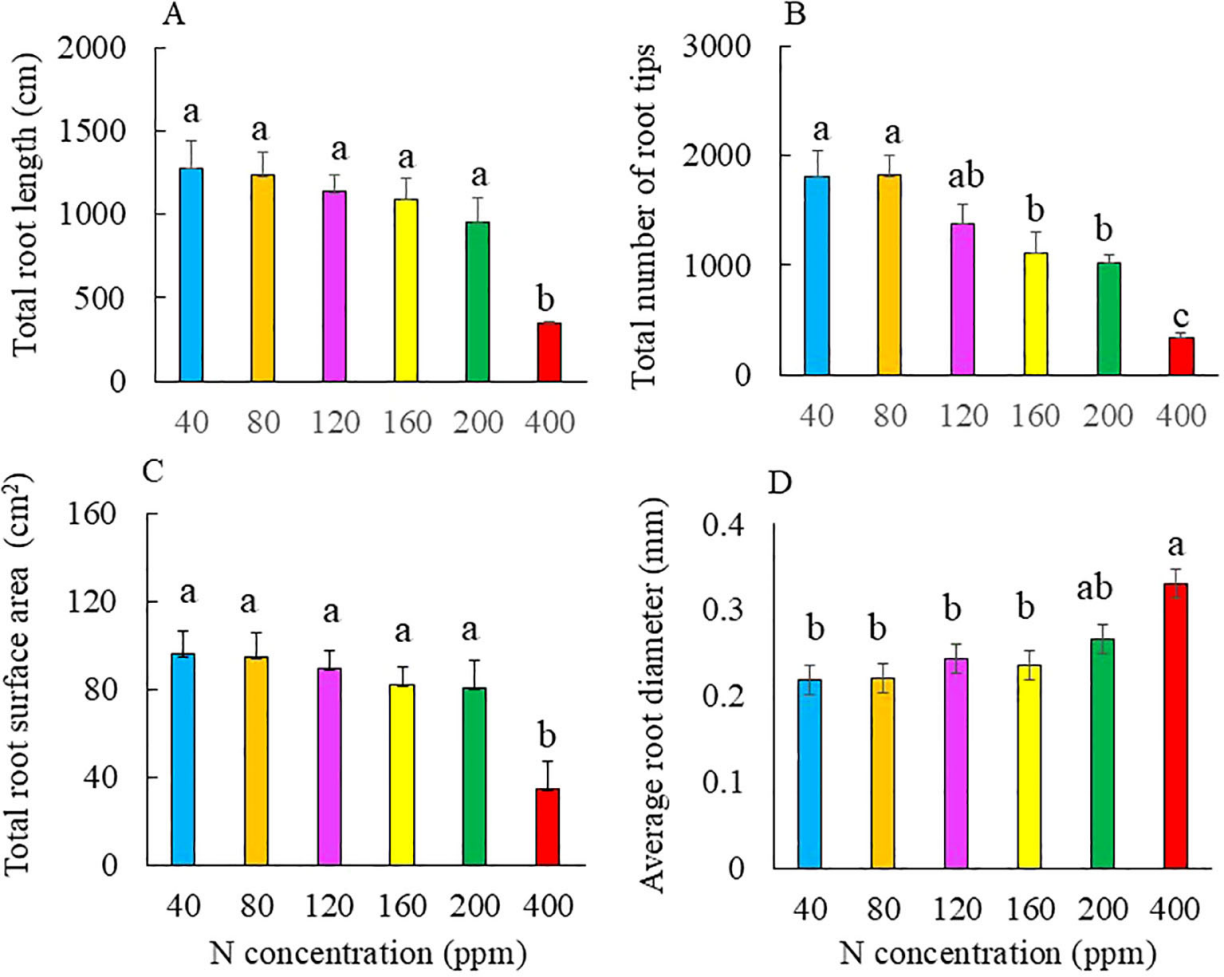
Total root length **(A)**, total number of root tips **(B)**, total root surface area **(C)**, and average root diameter **(D)** of Kai Lan plants grown with different NO_3_^−^ concentrations at harvest (5 weeks after transplanting). Values are means (± S.E.) where different letters indicate significant differences (*p* < 0.05) of four replicates as determined by Tukey’s multiple comparison tests.

Both total root length and surface area were significantly shorter and smaller for Kai Lan plants grown with 400 ppm N compared to those of the other plants. However, there were no significant differences in these two parameters among the Kai Lan plants supplied with 40 to 200 ppm N (**Figures 4A, C**). The total number of root tips was significantly smaller in plants with 200 and 400 ppm N compared to those of plants with 40 and 80 ppm N. There was no significant difference in the number of root tips among the plants supplied with 120, 160, and 200 ppm N, and plants grown with 40, 80, and 120 ppm N also had a similar total number of root tips (**Figure 4B**). The average root diameter was significantly thicker in plants supplied with 400 ppm N compared to those with 40 to 160 ppm N, but there was no significant difference between 200 and 400 ppm (**Figure 4D**).

### Photosynthetic pigments and photosynthetic light use efficiency

**Figure 5:**
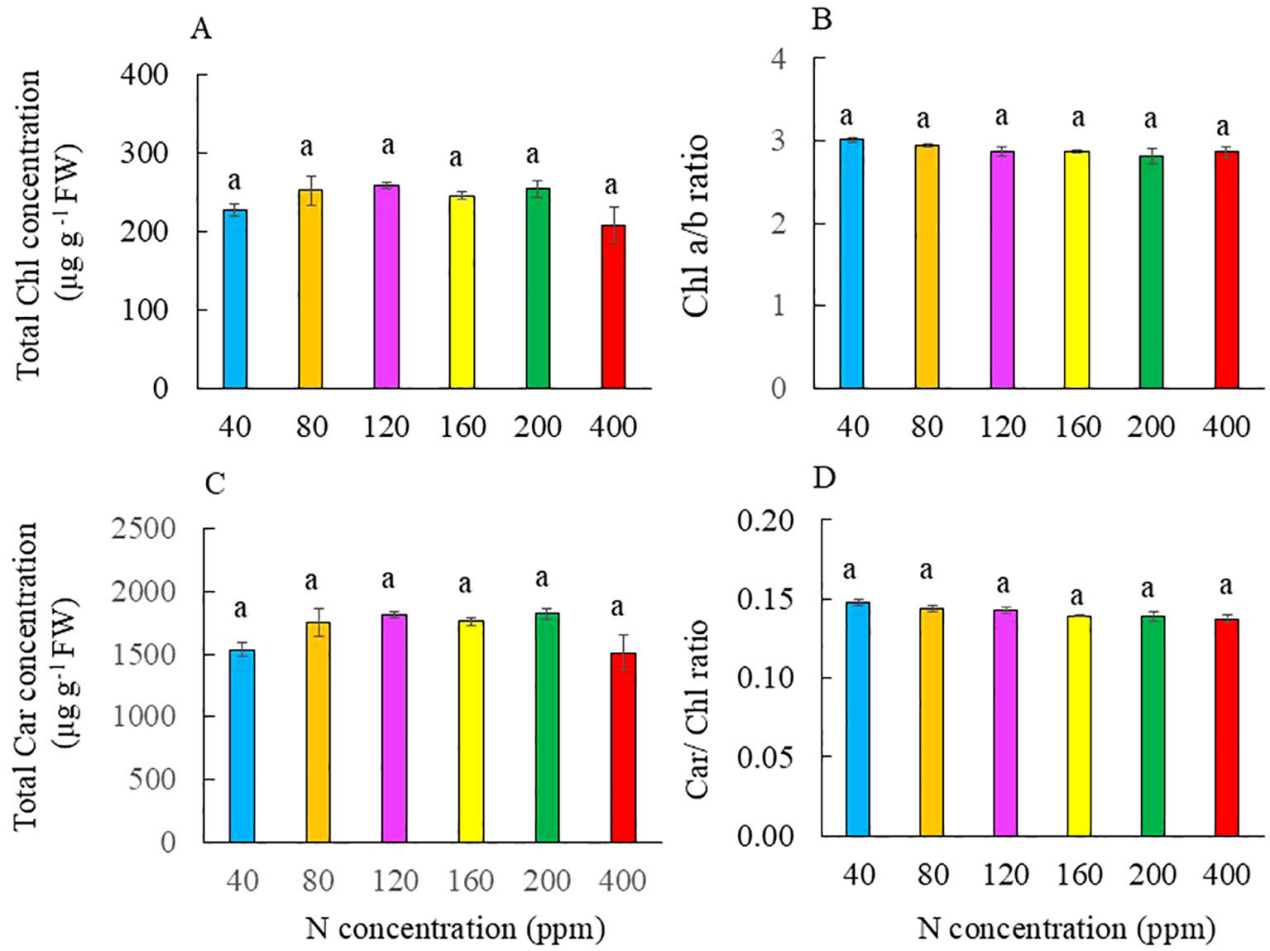
Total Chl concentration **(A)**, Chl a/b ratio **(B)**, total Car concentration **(C)**, and Car/Chl ratio **(D)** of Kai Lan plants grown with different NO_3_^−^ concentrations for 4 weeks. Values are means (± S.E.) of four replicates. All variables with the same letter indicate that the differences among the means are not statistically significant (*p* < 0.05) as determined by Tukey’s multiple comparison tests.

**Figure 6:**
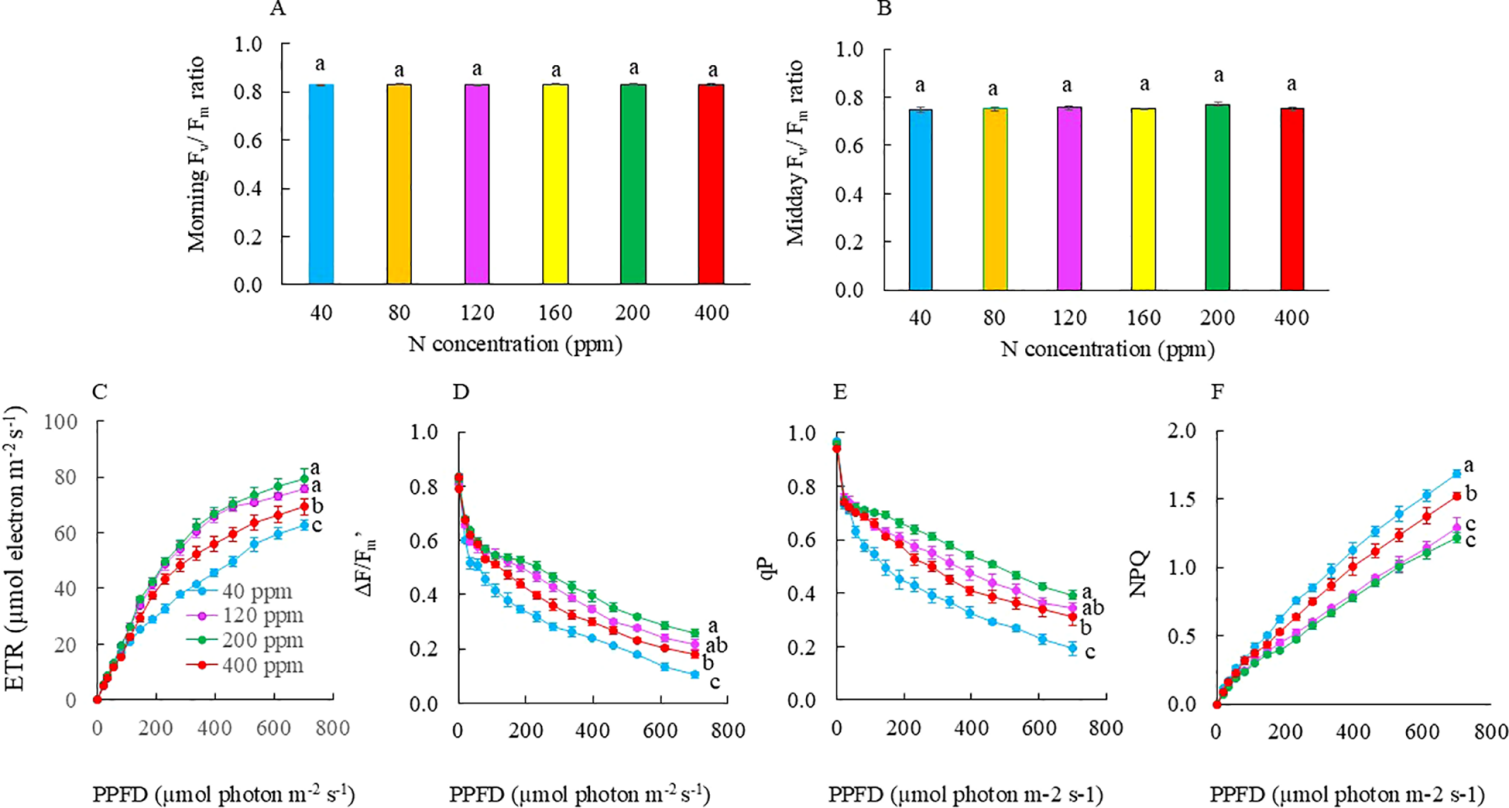
Morning F_v_/F_m_ ratio **(A)**, midday F_v_/F_m_ ratio **(B)**, ETR **(C)**, ΔF/F_m_′ **(D)**, qP **(E)**, and NPQ **(F)** of Kai Lan plants grown with different N concentrations for 4 weeks. Values are means (± S.E.) where different letters indicate significant differences (*p* < 0.05) of eight replicates for **(A, B)** and four replicates for **(C–F)**, as determined by Tukey’s multiple comparison tests.

There were no significant differences in total Chl (**Figure 5A**) and total Car concentrations (**Figure 5B**), Chl a/b ratio (**Figure 5C**), and Chl/Car ratio (**Figure 5D**) among the treatment groups. In the morning, the F_v_/F_m_ ratios of all the plants were greater than 0.8, ranging from 0.825 to 0.830, with no significant difference (**Figure 6A**). These results support the observation that all plants were healthy with no chronic photoinhibition. During midday, with increasing PPFD, all plants had their F_v_/F_m_ ratios slightly lower than 0.8 with their values ranging from 0.750 to 0.770, but no significant difference was observed among the treatment groups (**Figure 6B**). The light response curves of ETR and NPQ showed increasing trends (**Figures 6C, F**), while the light response curves of ΔF/F_m_′ and qP showed decreasing trends with increasing PPFD from 1 to 701 μmol m^−2^ s^−1^ for all the plants (**Figures 6D, E**). Measured at a PPFD of 701 μmol m^−2^ s^−1^, which is close to the maximal PPFD inside the greenhouse on sunny days, ETR, ΔF/F_m_′, and qP were significantly the lowest in Kai Lan plants grown with 40 ppm N followed by those grown with 400 ppm N. These three parameters were similar and significantly higher in Kai Lan plants grown with 120 and 200 ppm N compared to those of plants grown with 40 and 400 ppm N (**Figures 6C–E**). However, NPQ values were significantly the highest in plants grown with 40 ppm N followed by those with 400 ppm N and plants grown with 120 and 200 ppm N had the significantly lowest NPQ values (**Figure 6F**). There were no significant differences in ETR, ΔF/F_m_′, qP, and NPQ values among plants supplied with 80 and 160 ppm N compared to those of plants grown with 120 and 200 ppm N. For the clarity of **Figures 6C–F**, the results of ETR, ΔF/F_m_′, qP, and NPQ obtained from Kai Lan plants grown with 80 and 160 ppm N are not included.

### Photosynthetic gas exchange

**Figure 7:**
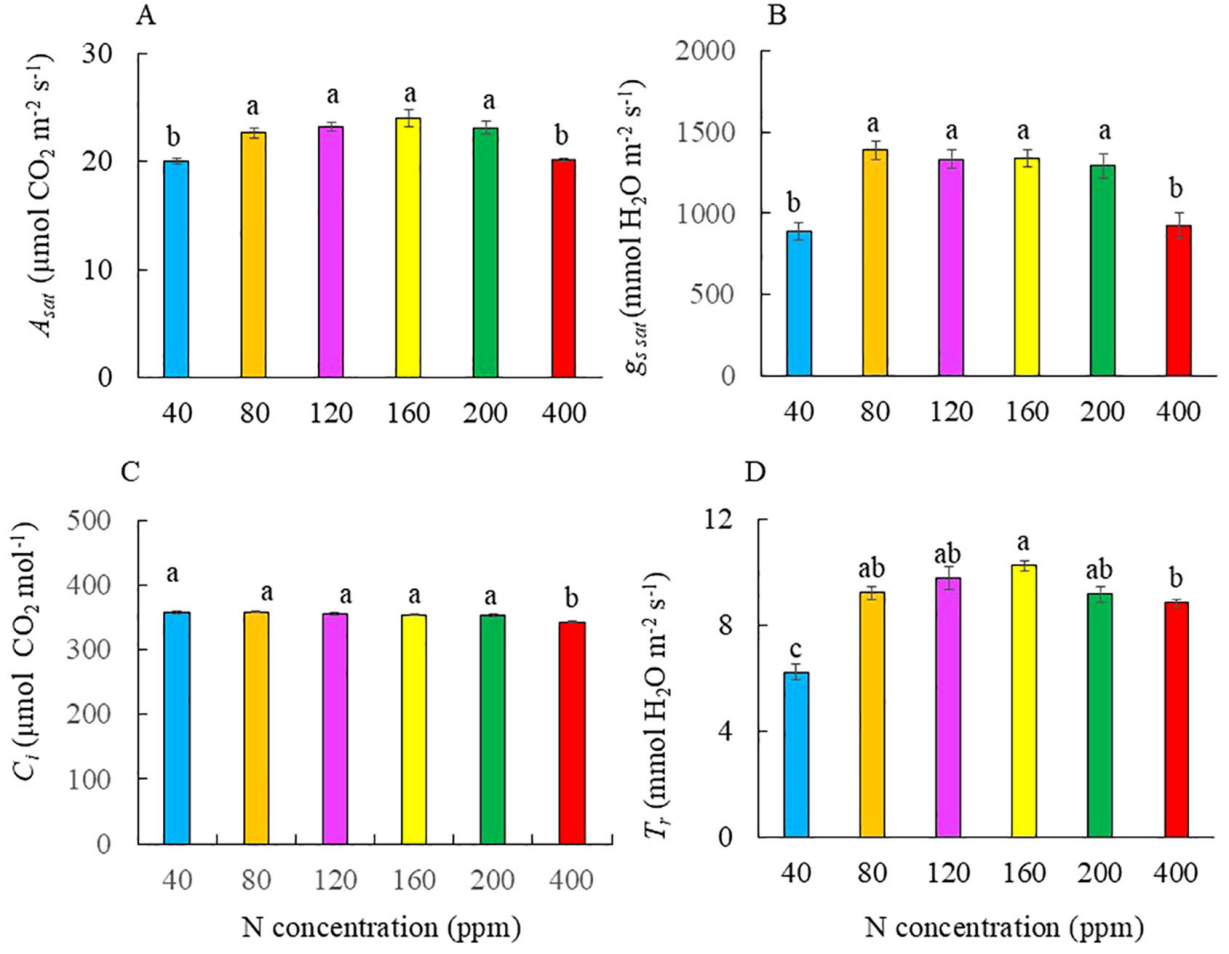
*A*_sat_**(A)**, *g*_s sat_**(B)**, *C*_i_**(C)**, and *T*_r_**(D)** of Kai Lan plants supplied with different N concentrations for 4 weeks. For each treatment, four measurements were made from four different leaves of four different plants. Values are means (± S.E.) where different letters indicate significant differences (*p* < 0.05) of four replicates as determined by Tukey’s multiple comparison tests.

There were no significant differences in *A*_sat_ and *g*_s sat_ for Kai Lan plants supplied with 80 to 200 ppm N. However, *A*_sat_ and *g*_s sat_ were significantly lower at 40 and 400 ppm N compared to those grown with 80, 120, 160, and 200 ppm N (**Figures 7A, B**). There was no significant difference in *C*_i_ among plants grown with 40 to 200 ppm N, but plants grown with 400 ppm N had a significantly lower value of *C*_i_ (**Figure 7C**). Kai Lan plants grown with 40 ppm N had the lowest *T*_r_ followed by plants grown with 400 ppm N. Kai Lan plants grown with 160 ppm N had the highest *T*_r_ (**Figure 7D**). However, there was no significant difference in *T*_r_ among Kai Lan plants grown with N from 80 to 200 ppm (**Figure 7C**).

### NO_3_^−^, TRN, leaf TSP, Rubisco protein, NUE, and NHI

**Figure 8:**
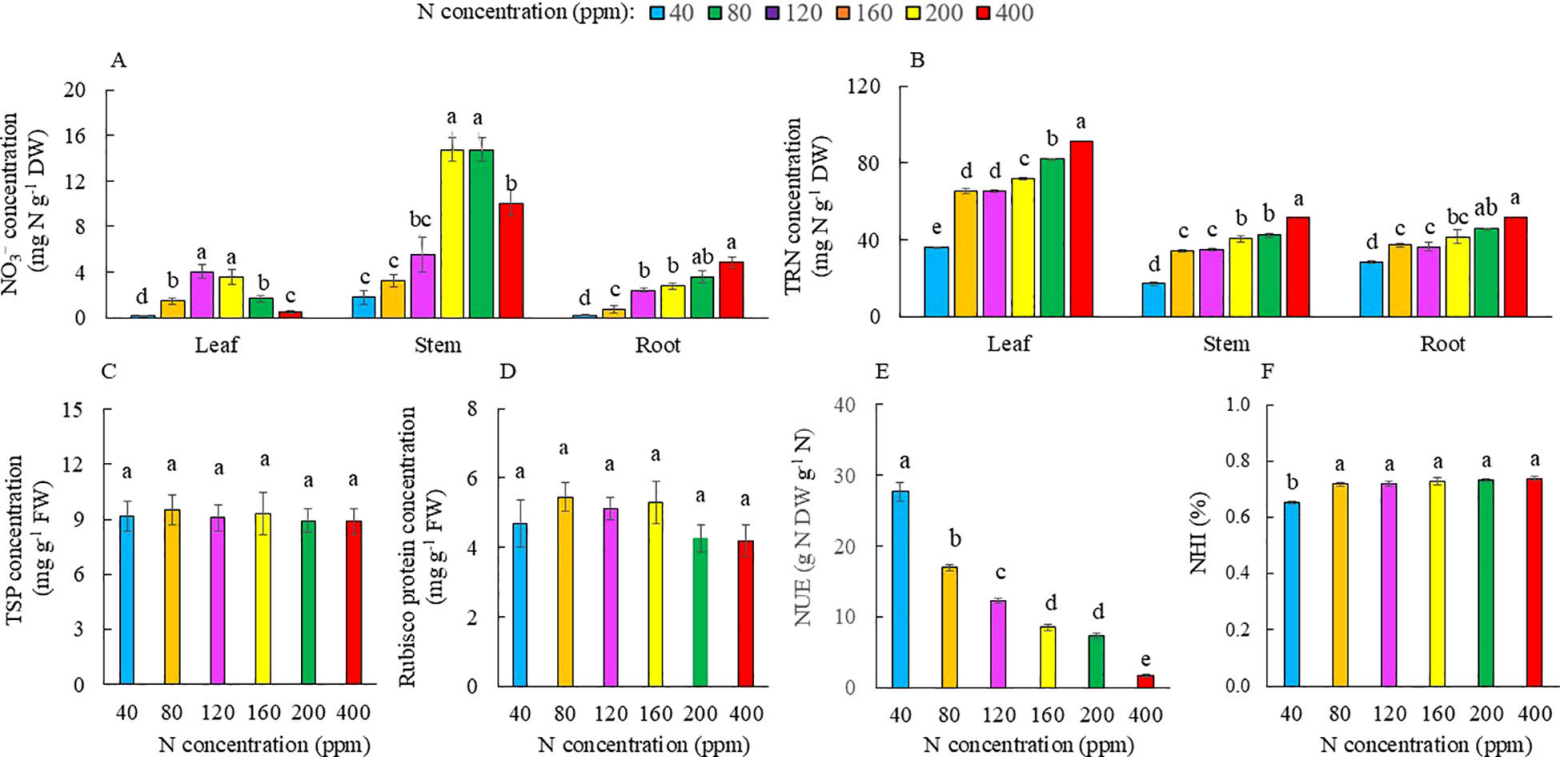
NO_3_^−^ concentration **(A)**, TRN concentration **(B)**, TSP concentration **(C)**, Rubisco protein concentration **(D)**, NUE **(E)**, and NHI **(F)** of Kai Lan plants grown with different N concentrations at harvest (5 weeks after transplanting). NO_3_^−^ and TRN were measured from the roots, stems, and leaves, while TSP and Rubisco were only measured from leaves. Values are means (± S.E.) where different letters indicate significant differences (*p* < 0.05) of four replicates as determined by Tukey’s multiple comparison tests.

Accumulation of NO_3_^−^ in the leaves of Kai Lan plants increased with increasing N supply from 40 to 120 ppm N, with a maximum accumulation from 120 to 160 ppm N, and decreased from 200 to 400 ppm N. Accumulation of NO_3_^−^ in the stems increased with increasing N supply from 40 to 160 ppm N with a maximum accumulation from 160 to 200 ppm N and decreased with 400 ppm N. In the root, accumulation of NO_3_^−^ increased with increasing N supply from 40 to 400 ppm N with the highest concentration of NO_3_^−^ at 200 and 400 ppm N, with no significant difference between 120 and 200 ppm N and between 40 and 80 ppm N (**Figure 8A**). As N availability increased from 40 to 400 ppm, TRN in the leaves of Kai Lan plants increased with the lowest TRN at 40 ppm N and the highest TRN at 400 ppm N. However, there was no significant difference in TRN concentration between 80 and 120 ppm N (**Figure 8B**). Similarly, there was no significant difference in stem TRN concentration between 80 and 120 ppm N as well as between 160 and 200 ppm N (**Figure 8B**). Root TRN concentration was also the lowest at 40 ppm and the highest at 400 ppm N. There was no significant difference in TRN concentration from 80 to 200 ppm N and between 200 and 400 ppm N (**Figure 8B**). There were no significant differences in TSP and Rubisco protein concentrations among the different treatments (**Figures 8C, D**). NUE, which is the yield per unit of fertilizer N applied, was the highest in plants supplied with 40 ppm N and decreased with increasing N supply with the lowest NUE at 400 ppm N (**Figure 8E**). There was no significant difference in NHI, the ratio of N in harvestable shoot divided by the total plant N, from 80 to 400 ppm N, but it was the lowest in plants grown at 40 ppm N (**Figure 8F**).

### Phytonutrients and dietary minerals

**Figure 9:**
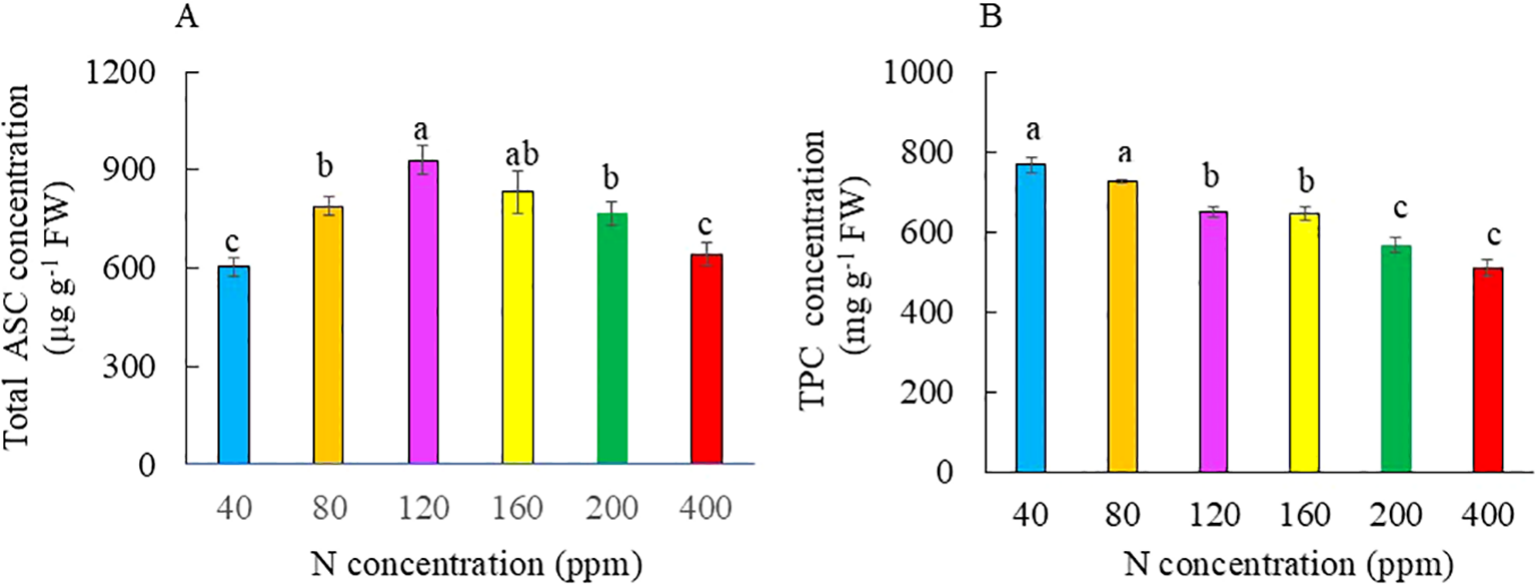
Total ASC concentration **(A)** and TPC concentration **(B)** of Kai Lan plants grown with different N concentrations at harvest (5 weeks after transplanting). Values are means (± S.E.) where different letters indicate significant differences (*p* < 0.05) of four replicates as determined by Tukey’s multiple comparison tests.

**Figure 10:**
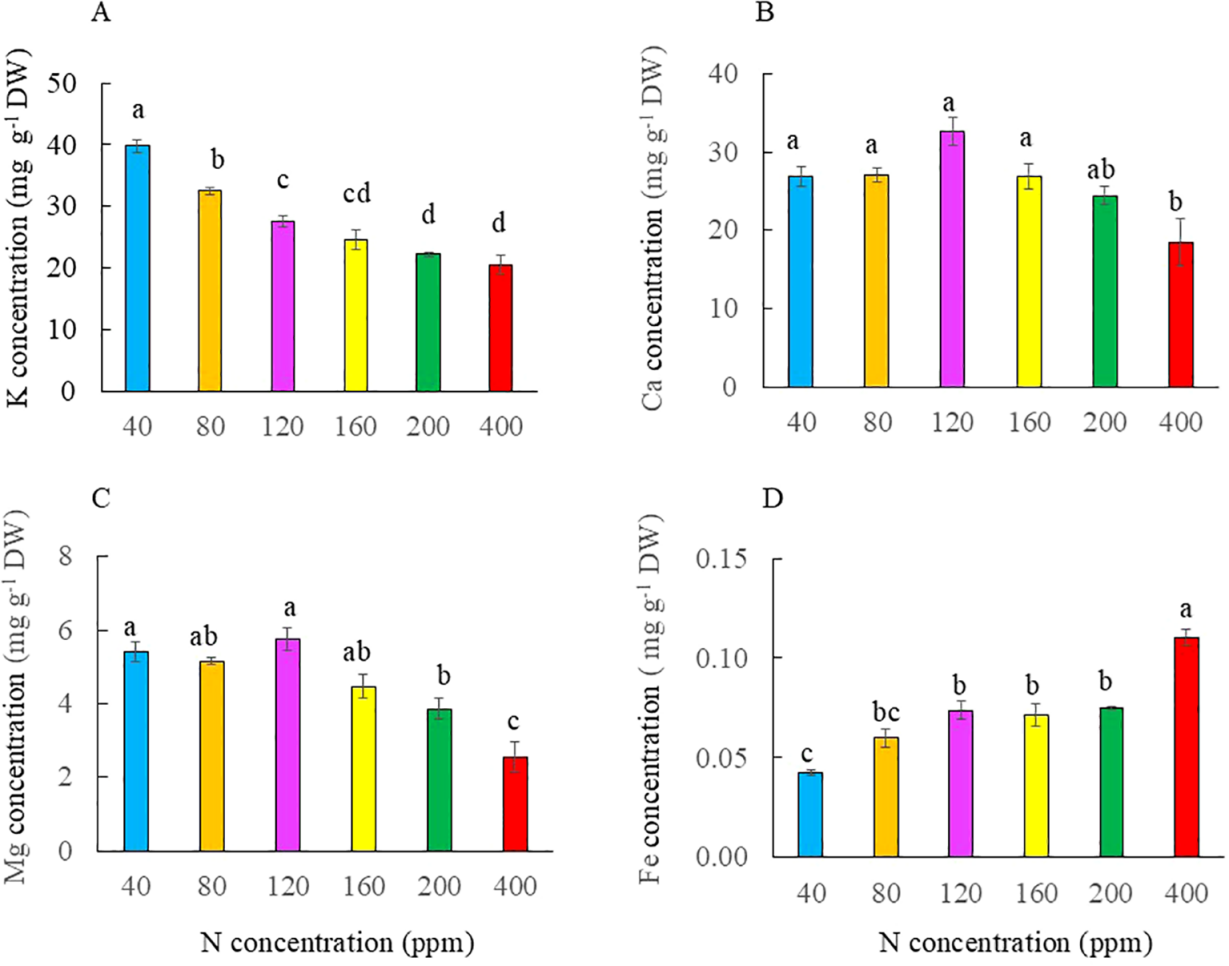
Concentrations of K **(A)**, Ca **(B)**, Mg **(C)**, and Fe **(D)** in the leaves of Kai Lan plants grown with different N concentrations at harvest (5 weeks after transplanting). Values are means (± S.E.) where different letters indicate significant differences (*p* < 0.05) of four replicates as determined by Tukey’s multiple comparison tests.

The total ASC concentration was significantly lower in plants grown with both the lowest and highest N of 40 ppm compared to that of the other plants. Plants grown with 120 ppm N had the highest ASC concentration, but there was no significant difference from those grown with 160 ppm N. Plants grown with 120 ppm N had significantly higher ASC concentrations than those grown with 80 and 200 ppm N (**Figure 9A**). Leaf total TPC concentration was significantly higher in plants grown with 40 to 160 ppm N than with 200 and 400 ppm N (**Figure 9B**). Kai Lan plants grown with 40 ppm N had the highest K concentration, followed by those with 80 ppm N. Plants grown with 400 ppm N had the lowest K concentration. There was no significant difference in K concentration between plants grown with 120 and 160 ppm N and among plants with 160, 200, and 400 ppm N (**Figure 10A**). Plants grown with 40 to 200 ppm N had similar Ca concentration, which was significantly higher compared to plants grown at 400 ppm N (**Figure 10B**). Plants grown with 400 ppm N had the lowest Mg concentration compared to all the other plants. However, there was no significant difference in Mg concentration between plants grown with 400 and 200 ppm N, between plants grown with 200 and 160 ppm N, and among plants grown with 120, 80, and 40 ppm N (**Figure 10C**). Fe concentration was significantly lower in 40 ppm N plants compared to those of plants grown from 80 to 400 ppm N, but there was no difference among plants grown with 80 to 200 ppm N. Plants grown with 400 ppm N had the highest Fe concentration (**Figure 10D**).

## Discussion

### Leaf growth, root and shoot productivity, and root morphology

In this study, although Kai Lan plants grown with 40 and 400 ppm N were much smaller compared to the others, all plants appeared healthy (**Figure 1**). Severe N deficiency (40 ppm) did not affect leaf initiation, but leaf expansion was inhibited (**Figure 2B**). Under N limitation, plants reduce the size of the mature leaf to maintain N concentration and photosynthetic capacity per unit leaf area in sorghum (Zhao et al., 2005), winter oilseed rape (Liu et al., 2018), and maize (Mu and Chen, 2021). However, Kai Lan plants grown with 80, 120, and 160 ppm N had similar TLA as plants with full N of 200 ppm N (**Figure 2B**). As mentioned earlier, in the Hoagland solution, 200 ppm N is required for a full strength of hydroponic solution. Under 80, 120, and 160 ppm N, Kai Lan plants were indeed grown with 38%, 57%, and 76% of full N, respectively. These results imply that leaf expansion of Kai Lan plants was only inhibited by severe N deficiency of 40 ppm N (19% full N). In our previous study with ice plant (*Mesembryanthemum crystallinum*), leaf number and TLA were significantly reduced in plants grown with 25% of full N. However, ice plants grown with ½ N and ¾ N had greater leaf number and TLA compared to those of the full N plants (He and Qin, 2021). Furthermore, the present study found that both severe limited (40 ppm) and excessive (400 ppm) N supply increased the thickness of the leaf, reflected by lower SLA (**Figure 2C**). The lower TLA and SLA of Kai Lan plants grown with deficient N resulted in lower light interception and thus reduced the whole plant carbon gain and biomass accumulation (Zhao et al., 2005; He et al., 2011a, 2014; Liu et al., 2018; Mu and Chen, 2021; Godoy et al., 2025). Excessive supply of N also reduced leaf growth and crop yield (Chen et al., 2004; He et al., 2014; Dong and Lin, 2020). In this study, only Kai Lai plants grown with severe N limitation of 40 ppm and excessive N of 400 ppm had significantly lower FW and DW of the leaves, stems, and shoots compared to those of plants grown with full N 200 ppm. However, reducing nitrogen supply to 80, 120, and 160 ppm N, Kai Lan plants had similar shoot FW and DW compared to those grown with full N of 200 ppm (**Figures 3A, B, D, E**). A common response for plants grown with limited N is to allocate more newly fixed carbon to the roots (Smolders and Merckx, 1992). In this study, Kai Lan plants grown with 40 ppm N had the highest root FW and DW (**Figures 3B, E**) and lower shoot/root FW and DW ratios (**Figures 3C, F**). Similar results were observed with two lettuce (*Lactuca sativa*) recombinant inbred lines (RILs; He et al., 2014), where plants supplied with −N (50% NO_3_^−^) and +N (125% NO_3_^−^) had a lower shoot/root DW ratio compared to plants supplied with full N (100% NO_3_^−^). The lower shoot/root ratio was due to the greater decrease in shoot FW compared to the decrease in root FW. In this study, N deficiency did not change root FW and DW compared to plants grown with full N of 200 ppm (**Figures 3B, E**). However, Kai Lan plants grown with 80 to 200 ppm N had similar but significantly higher shoot/root FW and DW ratios than those of plants grown with 40 ppm N, resulting from their lower shoot FW and DW. In the study with *Arabidopsis*, Gruber et al. (2013) also found that root biomass decreased comparatively less than the shoot, and the shoot/root ratio increased with decreasing N supply. Conversely, excess N has an inhibitory effect on root growth, resulting in a smaller root system (**Figure 1**), with lower root FW and DW at 400 ppm N (**Figures 3B, E**) and a higher shoot/root FW and DW ratio (**Figures 3C, F**). The negative effects of excessive N on root biomass accumulation were reported by others (Okushima et al., 2011; Niu et al., 2024). In view of the growth traits, 80 to 120 ppm N seems to supply sufficient N for comparative crop yield compared to 200 ppm N plants, with 120 ppm N being the most optimal (the highest plant biomass and enhanced nutritional quality; **Figures 9**, **10**).

N availability not only affects root biomass accumulation but also root morphology (Lopez et al., 2023). Gruber et al. (2013) reported that the total root length of *Arabidopsis* increased by 48% under moderate N deficiency but decreased under the most severe N deficiency. In this study, N deficiency did not affect the total root length and total surface area compared to those grown with full N of 200 ppm (**Figures 4A, C**) until N deficiency reached the most severe 40 ppm, resulting in the longest root and the largest root surface area. Compared to a full N supply of 200 ppm, Kai Lan plants grown with 40 and 80 ppm also had significantly higher total number of root tips (**Figure 4B**), indicating that N deficiency promoted lateral root development. A similar result was reported by López-Bucio et al. (2003) in *Arabidopsis*. A larger root surface area under N (NO_3_^−^) limitation was mainly due to the development of lateral roots (Sun et al., 2017; Liu et al., 2020; Pélissier et al., 2021). In our previous study with two lettuce RILs, decreases in total root length, total root surface area, and total number of root tips were observed in heat-sensitive (HS) RIL under both −N (50% NO_3_^−^) and +N (125% NO_3_^−^) compared to plants supplied with full N (100% NO_3_^−^). However, N availability did not affect the root morphology of heat-resistant (HR) RIL (He et al., 2014). Kai Lan plants grown with deficient N had a similar average root diameter as those of full N of 200 ppm (**Figure 4D**). The relationship between average root diameter with N supply manipulation appears to be species-specific (He et al., 2014; Lopez et al., 2023). In this study, excessive N supply with 400 ppm N resulted in significantly lower total root length, total number of root tips, and total root surface area but higher average root diameter compared to those with full N or moderate insufficient N (**Figure 4**). Okushima et al. (2011) reported that a high concentration of NO_3_^−^ supply to *Arabidopsis* suppressed lateral root formation and root elongation. This also reaffirms the resource optimization theory, where plants allocate fewer resources to the roots when there is an increase in nutrient availability (Ågren and Franklin, 2003). However, according to Saiz-Fernández et al. (2017), the inhibition of high NO_3_^−^ on root growth is not triggered by the external NO_3_^−^ concentration but by some internal factors such as ACC (1-aminocyclopropane-1-carboxylic acid) and its product, ethylene.

### Photosynthetic pigments, photosynthetic light use efficiency, and photosynthetic gas exchange

Limiting N is expected to reduce Chl production (Mu et al., 2016; Mu and Chen, 2021). Grown under limitation N, Chl concentration decreased in both C_3_ crops such as cucumber (Zhao et al., 2015), soybean, and rice as well as C_4_ crops including maize and sorghum (Mu and Chen, 2021). However, in this study, N deficiency and excessive N did not affect total Chl and Car concentrations and Chl a/b and Car/Chl ratios (**Figure 5**). In the study with the two lettuce RILs, we also reported that all plants had similar levels of total Chl and Chl a/b ratio regardless of N availability (He et al., 2014). In this study, under the most severe limitation N of 40 ppm, Kai Lan plants reduced leaf area (**Figure 2A**) and maintained adequate leaf TRN concentration, approximately 4% (**Figure 8B**). The small total leaf area compensates for the lower N availability, which may be a strategy used by plants to cope with N limitation. The smaller the leaf area, the less N is needed to maintain Chl concentration. Saiz-Fernández et al. (2017) also found that excessive NO_3_^−^ supply had similar total Chl and Car concentrations in maize plants grown with low NO_3_^−^ for 45 days.

Within the chloroplast thylakoid membrane, N is needed to synthesize proteins involved in electron transport and photosynthetic enzymes associated with photophosphorylation (Mu et al., 2016) and light energy allocation (Huang et al., 2004; Mu and Chen, 2021). In this study, all plants had similar F_v_/F_m_ ratio in the morning (**Figure 6A**), indicating that no chronic photoinhibition occurred in any plants (Björkman and Demmig, 1987). Midday F_v_/F_m_ ratio slightly decreased with increased PPFD, and there was no significant difference among the different treatments (**Figure 6B**). These results indicate that neither N deficiency nor excessive N results in photodamage of the PSII reaction centers and severe D1 protein degradation for Kai Lan plants. Kanash et al. (2023) reported that no significant changes in F_v_/F_m_ ratio were found in lettuce after 28 days of reduced N treatment. In rice and winter wheat, N deficiency decreased the F_v_/F_m_ ratio (Mu and Chen, 2021). However, Peng et al. (2021) reported that the effects of N application rate on F_v_/F_m_ ratio depend on the growth stage and other environmental factors. To keep the clarity of the figures, the light response curves of ETR, ΔF/F_m_′, qP, and NPQ were only shown for Kai Lan plants grown with 40, 120, 200, and 400 ppm N (**Figures 6C–F**), as 80 and 160 ppm N plant responses were statistically similar to those of 120 and 200 ppm N plants under the same PPFDs. Kai Lan grown with 40 ppm N had the lowest values of ETR, ΔF/F_m_′, and qP under a PPFD of 146 μmol m^−2^ s^−1^ or above. However, 40 ppm N plants had the highest NPQ level, followed by those grown with 400 ppm N and the lowest NPQ values in both 120 and 200 ppm N plants (**Figure 6F**). These results were similar to rice plants in which ETR decreased and NPQ increased after being subjected to N deficiency (Huang et al., 2004). In this study, the F_v_/F_m_ ratio was not affected, which could be due to the higher NPQ at 40 ppm N, dissipating excess light energy as heat. Thus, the PSII center was not damaged (Tikkanen et al., 2014), supporting that there was no chronic photoinhibition for all treatments (Björkman and Demmig, 1987). At the lowest N supply of 40 ppm, Kai Lan plants had 3.6% leaf TRN, which was approximately 40% to 55% compared to those of the other plants (**Figure 8B**). This could be due to decreased N allocation for protein synthesis related to light-harvesting and electron transport such as the content of PSI, PSII, cytochrome b6f (*Cyt b6f*) complex, and ATP synthase and photosynthetic enzymes (Mu and Chen, 2021). Some studies have proved that there is a positive correlation between the content of *Cyt b6f* and Rubisco (Salesse-Smith et al., 2018). In this study, all Kai Lan plants grown with different N had a similar level of Rubisco protein (**Figure 8D**). Yamori et al. (2011) reported that a reduction in *Cyt b6f* content or ATP synthase in tobacco had lower ETR and higher NPQ, suggesting that *Cyt b6f* could be the key rate-limiting step for electron transport. However, it is unclear why excessive N supply with 400 ppm resulted in lower ETR, ΔF/F_m_′, and qP and higher NPQ compared to full N supply of 200 ppm and those grown with 120 ppm N. In the study with Chinese ginseng (*Panax notoginseng*), Cun et al. (2021) reported that under excessive N, photosynthetic capacity might be primarily inhibited by the inactivated Rubisco, resulting in photodamage to the donor side of the PSII oxygen-evolving complex.

Mu et al. (2018) reported that limited N promotes the biosynthesis of ABA, resulting in stomata closure and leading to decreased *C*_i_. With lower *g*_s sat_ of Kai Lan plants grown with severe N deficiency of 40 ppm (**Figure 7B**), *C*_i_ was expected to decrease. However, N deficiency did not result in lower *C*_i_ compared to plants grown with full N of 200 ppm (**Figure 7C**). This suggests that the concentration of CO_2_ available inside the leaf is unlikely to be the limiting factor for decreased *A*_sat,_ but due to non-stomatal factors such as reduced *Cyt b6f* complex discussed earlier under N limitation (Salesse-Smith et al., 2018; Mu and Chen, 2021). Under severe N limitation of 40 ppm N, decreased *A*_sat_ could also be due to reduced Rubisco concentration (Evans and Clarke, 2019; Mu and Chen, 2021; Godoy et al., 2025). However, in this study, all Kai Lan plants had a similar level of Rubisco protein (**Figure 8E**), indicating that Rubisco concentration was not the limiting factor as a result of N supply. Grown at 400 ppm N, *A*_sat_ was lower than in plants grown with 80 to 200 ppm N (**Figure 7A**). A likely explanation is the reduced *g*_s sat_ (**Figure 7B**), leading to a lower *C*_i_ (**Figure 7C**) and *T*_r_ (**Figure 7D**). Under excessive N conditions, decreased *A*_sat_ could also occur due to the possible competition for energy and reductants between N and C metabolism (Saiz-Fernández et al., 2017).

### NO_3_^−^, TRN, leaf TSP, Rubisco protein, NUE, and NHI

Chinese broccoli, Kai Lan, is one of the leafy vegetable crops that can accumulate large amounts of NO_3_^−^ (Dich et al., 1996). Leaf NO_3_^−^ concentration increased with increasing N supply from 40 to 120 ppm N and reached maximum accumulation from 120 to 160 ppm N and decreased from 200 to 400 ppm N. Kai Lan stems had the highest NO_3_^−^ concentration compared to both leaves and roots (**Figure 8A**). Expressed in terms of FW, the highest NO_3_^−^ level was observed in the stems of plants grown under 160 and 200 ppm N (1,390 to 1,400 mg^−1^ FW), while the leaves had much lower NO_3_^−^ concentrations (340 and 160 mg kg^−1^ FW, **Figure 8A**). According to Luo et al. (2022), 25% of 264 fresh vegetables randomly collected in the farmers’ markets had a NO_3_^−^ concentration reaching the critically contaminated level of >1,440 mg/kg FW. The thick, green succulent stems of Kai Lan plants grown with 160 and 200 ppm N make up more than 50% of the edible shoot (**Figures 1**, **2A**), with NO_3_^−^ concentration almost reaching the lower critical range of 1,440 mg/kg FW. NO_3_^−^ has low toxicity to humans. However, the risk of NO_3_^−^ for human health is the reduction of NO_3_^−^ to nitrite (NO_2_^−^), which, when consumed in excessive amounts, can lead to the formation of carcinogenic nitrosamines (Ciriello et al., 2021). Thus, it would be beneficial for consumers to reduce the concentration in edible tissues by lowering NO_3_^−^ in the nutrient supply. Based on our results, it is recommended to supply Kai Lan plants with 80 to 120 ppm N with NO_3_^−^ concentration of 300 to 500 mg/g FW in the stems, which is below the critical contaminated level.

NO_3_^−^ absorbed is reduced to NH_4_^+^ during NO_3_^−^ assimilation, which is then used to form different organic nitrogen compounds including amino acids and proteins. In this study, TRN concentrations indicate the N status of the leaves, stems, and roots of Kai Lan plants grown with different levels of N (**Figure 8B**). For healthy plants, N constitutes 3%–4% of their aboveground tissues (Uchida, 2000). In this study, plants grown with the lowest N level of 40 ppm had TRN concentrations of 3.61% and 1.63% in their leaves and stems, respectively, while 80~400 ppm N plants had leaf TRN ranging from 3.42% to 9.13%. These results suggest that all plants had TRN greater than 3% in both the leaves and stems apart from the stems of Kai Lan plants grown with 40 ppm N, which had a TRN concentration of 1.63%. The TRN concentrations in the leaves were higher compared to both the stems and roots, indicating a higher NO_3_^−^ assimilation efficiency in the leaves (Smirnoff and Stewart, 1985). TRN concentrations were the highest in the leaves, stems, and roots of Kai Lan plants grown with 400 ppm N. With increased NO_3_^−^ supply, the rate of NO_3_^−^ assimilation and nitrate reductase activity (NRA) increases (Foyer et al., 2003). The assimilation of NO_3_^−^ requires 2-oxoglutarate, a major source of carbon skeleton, resulting in decreased carbon availability for other metabolic processes. This higher energy demand for NO_3_^−^ assimilation at 400 ppm N likely resulted in stunted leaf growth (**Figure 2**) and decreased shoot and root biomass accumulation (**Figure 3**). It is well known that approximately 50% leaf N would be invested as soluble protein, of which approximately 50% would be assembled as Rubisco protein within the leaf (Mu and Chen, 2021). It is interesting to note that there was no significant difference in both TSP and Rubisco protein across the different N treatments (**Figures 8C, D**). These results imply that lower *A*_sat_ (**Figure 7A**) in Kai Lan plants grown with severe N limitation of 40 ppm and excessive N of 400 ppm is not due to the Rubisco concentration. Instead, they may mainly result from lower *g*_s sat_ (**Figure 7B**). On the other hand, under limited and excessive N, lower *A*_sat_ could also be partly due to lower ETR, ΔF/F_m_′, and qP and higher NPQ (**Figures 6C–F**).

For soilless vegetable farming, NUE can be used for environmental and economic objectives of minimizing N losses and the negative impact on surrounding water (Congreves et al., 2021). In this study, the higher the N supply, the lower the NUE (**Figure 8E**). Hence, it is recommended to provide Kai Lan plants with N concentration of 120 ppm N instead of 160 ppm N, as the plants grew well with a lower accumulation of NO_3_^−^ in the shoots (leaves + stem; **Figure 8A**) but better nutritional quality (**Figures 9**, **10**) without yield penalty (**Figures 2**, **3**). It is not necessary to provide Kai Lan plants with N concentration of more than 160 ppm N, as there was no significant difference in NHI (**Figure 8F**), despite the lower NUE. In this study of the leafy vegetable, NHI is the fraction of plant N in the harvested shoot, while NUE is the yield per unit N supplied (Congreves et al., 2021). The excess, unutilized N that is not absorbed by the plants increases the NO_3_^−^ runoff into water bodies, leading to eutrophication, harming aquatic ecosystems (Fageria and Baligar, 2005).

### Nutritional quality

It was reported that N limitation increased ASC (Zhao et al., 2015; Zhang et al., 2016) and TPC concentrations (Kováčik et al., 2007), whereas excessive N lowered both ASC and TPC concentrations (Mozafar, 1993; Lee and Kader, 2000; Stefanelli et al., 2010). In this study, Kai Lan grown with severe N limitation of 40 ppm and excessive N had the lowest total ASC concentration. Plants grown with 120 ppm N had significantly higher total ASC concentration compared to full N supply of 200 ppm (**Figure 9A**). These results indicate that the effects of N deficiency on the accumulation of ASC depend on the degree of N limitation. In the review article, Mozafar (1993) summarized that N overfertilization increased the concentration of NO_3_^−^ and simultaneously decreased ASC in many different fruits and vegetables including *Brassica* species. Others also reported that N overfertilization resulted in a significant reduction in ASC in broccoli and cauliflower (Lee and Kader, 2000), yellow grape tomatoes, capsicum, and leeks (Stefanelli et al., 2010). In this study, Kai Lan grown with excessive N of 400 ppm had significantly lower ASC concentration compared to those grown with 80 to 200 ppm N. This is likely because more energy is used for N assimilation when plants are grown with excessive N, resulting in less available energy to produce ASC (Saiz-Fernández et al., 2017). Compared to full N of 200 ppm, Kai Lan grown with 40 and 80 ppm N had the highest TPC concentration, followed by those of plants with 120 and 160 ppm N (**Figure 9B**). Grown with low N, high accumulation of TPC is likely due to an increase in phenylalanine ammonia-lyase (PAL) activity. PAL is situated at a branch point between primary and secondary metabolism, and it catalyzes an important regulatory step in the formation of many phenolic compounds (Kováčik et al., 2007). In contrast, high N supply at 400 ppm has the lowest TPC concentration, which is similar to that of full N plants with 200 ppm N (**Figure 9B**). It was reported that higher N supply decreased PAL activity and TPC concentration (Strissel et al., 2005). The nutritional quality of vegetables includes not only phytonutrients but also dietary minerals (Dias, 2012). Kai Lan is a rich source of dietary minerals such as K, Ca, Mg, and Fe, which are pivotal to human health (Zhang et al., 2025). N levels in the growth medium influence the morphology and architecture of the roots, along with the ability of the roots to absorb specific dietary minerals, determining the concentrations of dietary minerals (Pélissier et al., 2021; Lopez et al., 2023). In this study, accumulation of K, Ca, and Mg was enhanced in plants grown with N deficiency compared to those with full N of 200 ppm. However, Kai Lan supplied with excessive N and full N had similar lower levels of K, Ca, and Mg compared to those with lower levels of N (**Figures 10A–C**). Supplying Kai Lan plants with low N did not affect their root growth and development compared to full N, while excessive N supply retarded the growth and development of Kai Lan roots (**Figures 4A–C**). The higher mineral accumulation in Kai Lan plants supplied with low N could be due to their well-developed roots. For plants grown with severe N limitations, the higher concentrations of minerals may also be due to the reduced biomass (Li et al., 2022). However, plants grown with excessive N did not accumulate higher K, Ca, and Mg in their smaller plants. K and Mg are necessary elements for photosynthesis. In addition, K is required to regulate the opening and closing of stomata (Tränkner et al., 2018). The high accumulation of K in Kai Lan plants supplied with the lowest levels of 40 ppm suggests that their lower *A*_sat_ and *g*_sat_ (**Figures 7A, B**) were not caused by K and Mg deficiency. A lower Ca concentration in Kai Lan plants grown under excessive N of 400 ppm N plants could be partially responsible for their poorer root development (**Figure 4**), as low Ca levels can reduce the structural integrity of cells, reducing cell expansion (Hepler, 2005). Fe concentration in plants grown with 40 ppm N was significantly the lowest, while plants grown with the other levels of N deficiency from 80 to 160 ppm had similar levels of Fe as those of plants grown with the full N level of 200 ppm. Kai Lan plants grown at 400 ppm N had significantly the highest Fe concentration (**Figure 10D**), which aligns with the studies done where increasing N supply enhances the abundance of Fe transporter proteins (Kutman et al., 2011). Although supplying Kai Lan plants with excessive N of 400 ppm N increases the concentration of Fe, it is not recommended as excessive N significantly reduces K, Ca, and Mg concentrations, with a heavy yield penalty. It also lowers phytonutrients such as ASC and TPC. Conversely, the lowest N of 40 ppm reduced the Fe concentration in Kai Lan plants (**Figure 10D**). Thus, it is recommended to supply Kai Lan plants with 120 ppm N to ensure their nutritional quality is maintained without compromising yield, especially in the edible parts of the plant.

## Conclusion

Both severely limited (40 ppm) and excessive (400 ppm) N reduced the photosynthetic rates, yield, and nutritional quality of Kai Lan plants grown hydroponically. Excessive N also had a negative impact on root growth and development. Based on the results of this study, 80 to 160 ppm N maintained yield and photosynthetic performance comparable to full N of 200 ppm while improving nutritional quality and reducing NO_3_^−^ Considering that productivity, NUE, and NHI are the most favorable at 120 ppm N, it would be recommended that farmers supply Kai Lan plants with 120 ppm N when grown hydroponically. This ensures the production of high-quality Kai Lan plants without a yield penalty while minimizing both production cost in nutrient provision and post-harvest environmental pollution. However, this recommendation based on hydroponically grown Kai Lan in the tropical greenhouse may not be directly applied to other growth systems (e.g., soil), climates, or cultivars.

## Data Availability

The raw data supporting the conclusions of this article will be made available by the authors, without undue reservation.
