# Supplementary material for: Reduced nitrogen availability in hydroponically grown Chinese broccoli does not affect photosynthetic performance and yield while enhancing nitrogen use efficiency and nutritional quality
Source: Front Plant Sci. 2026 Jan 21;16:1745794. doi: 10.3389/fpls.2025.1745794 (PMC12869317; doi:10.3389/fpls.2025.1745794)
Supplement: Supplementary file 1 [file Table1.docx]

TABLE S1 Composition of the different nutrient solutions used for the different nitrogen treatments.

| Ingredients | N fertilizer dosages (ppm or mg/L) | | | | | |
| --- | --- | --- | --- | --- | --- | --- |
|  | 40 | 80 | 120 | 160 | 200 | 400 |
| KNO_3_ (mg/L) | 34.000 | 34.000 | 34.000 | 34.000 | 34.000 | 34.000 |
| K_2_SO_4_ (mg/L) | 150.000 | 150.000 | 150.000 | 150.000 | 150.000 | 150.000 |
| NaNO_3_ (mg/L) | 0.000 | 0.000 | 250.00 | 360.000 | 360.000 | 360.000 |
| NH_4_NO_3_ (mg/L) | 98.000 | 212.000 | 212.000 | 277.000 | 391.000 | 964.000 |
| MgSO_4_·0H2O (mg/L) | 150.000 | 150.000 | 150.000 | 150.000 | 150.000 | 150.000 |
| C_10_H_12_N_2_NaFeO_8_ (mg/L) | 18.000 | 18.000 | 18.000 | 18.000 | 18.000 | 18.000 |
| MnSO_4_·1H_2_O (mg/L) | 5.500 | 5.500 | 5.500 | 5.500 | 5.500 | 5.500 |
| ZnSO_4_·7H_2_O (mg/L) | 1.320 | 1.320 | 1.320 | 1.320 | 1.320 | 1.320 |
| CuSO_4_·5H_2_O (mg/L) | 0.496 | 0.496 | 0.496 | 0.496 | 0.496 | 0.496 |
| H_3_BO_4_ (mg/L) | 0.217 | 0.217 | 0.217 | 0.217 | 0.217 | 0.217 |
| MoO_3_ (mg/L) | 0.131 | 0.131 | 0.131 | 0.131 | 0.131 | 0.131 |
| CaSO_4_·2H_2_O (mg/L) | 217.000 | 217.000 | 217.000 | 217.000 | 217.000 | 217.000 |
| H_3_PO_4_ (μL/L) | 60.000 | 60.000 | 60.000 | 60.000 | 60.000 | 60.000 |
